# Endométriome ombilical: à propos d’un cas et revue de la littérature: Umbilical endometrioma: a case report and literature review

**DOI:** 10.11604/pamj.2018.29.22.14520

**Published:** 2018-01-11

**Authors:** Omar Sow, William Valentin, Diouf Cheikh, Barboza Denis, Faye Samba Thiapato, Diallo Ibrahima, Gueye Serigne Modou Kane

**Affiliations:** 1 Service de Chirurgie Générale, Hôpital de La Paix de Ziguinchor, Sénégal; 2 Service de Gynéco-obstétricale, Hôpital de la Paix de Ziguinchor, Sénégal; 3 Service de Chirurgie Générale, Centre Hospitalier Régional de Ziguinchor, Sénégal; 4 Service Anesthésie-Réanimation, Hôpital de la Paix de Ziguinchor, Sénégal

**Keywords:** Endométriome, ombilicale, chirurgie, Endometrioma, umbilical, surgery

## Abstract

L’endométriose se définit comme l’implantation de tissu endométrial en dehors de la cavité utérine. Elle touche environ 10% des femmes en âge de procréer. La localisation ombilicale est rare et la physiopathologie mal connue. Nous rapportons le cas d’une patiente de 42 ans, nulligeste, aux antécédents de myomectomie 5 ans auparavant, qui présentait une douleur cyclique avec une masse ombilicale dont le diagnostic était en faveur d’un endométriome ombilical, confirmée par l’étude histologique de la pièce de biopsie de la masse. Le traitement a consisté à une exérèse large de la masse associée à une exploration du pelvis et une plastie ombilicale.

## Abstract (en)

Endometriosis is defined as the implantation of endometrial tissue outside of the uterine cavity. It affects approximately 10% of women of childbearing age. Umbilical endometriosis is rare and its pathophysiology is poorly known. We report the case of a 42-year old nulliparous female patient with a 5-year history of myomectomy, presenting with cyclic pain associated with umbilical mass. The diagnosis of umbilical endometrioma was made and confirmed by the histological examination of the surgical specimen. Treatment was based on wide excision of the mass associated with pelvis exploration and umbilical plasty.

## Introduction

L’endométriose est définie par la présence d’épithélium endométrial avec stroma en dehors de la cavité utérine. Sa prévalence est estimée à environ 10% de la population féminine [1, 2]. Elle affecte les femmes en période d’activité génitale. Les principales localisations de l’endométriose sont par ordre de fréquence: pelvienne (80 à 90%), digestive (5 à 15%) et urinaire (2 à 4%). La localisation ombilicale n’a été que rarement décrite dans la littérature. Elle survient au niveau d’une cicatrice chirurgicale chez des femmes précédemment opérées [3]. Le terme d’endométriome est utilisé quand l’endométriose forme une masse bien limitée. A travers un cas d’endométriome ombilical rapporté, nous partageons une revue de la littérature sur la question.

## Patient et observation

**Figure 1:**
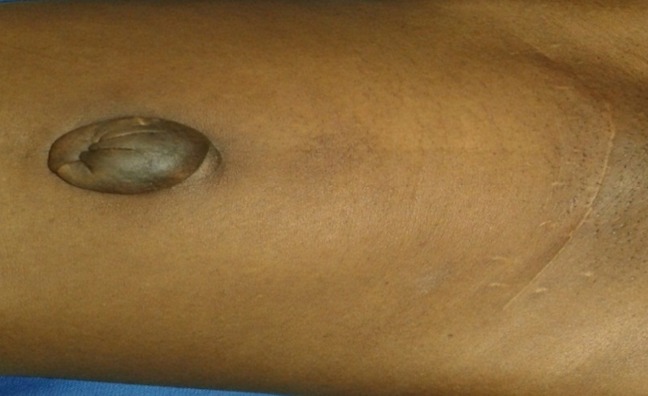
Masse ombilicale et cicatrice de Pfannenstiel

**Figure 2:**
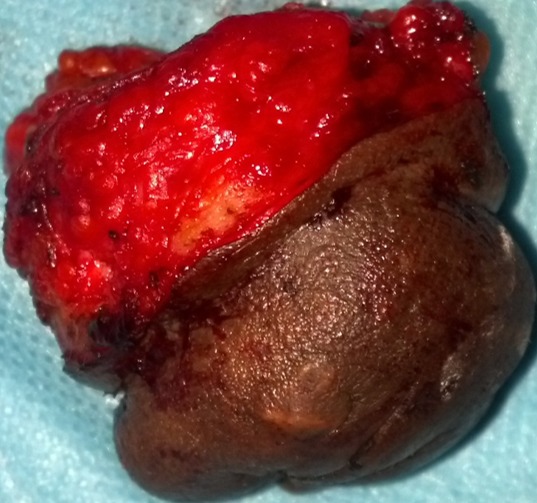
Pièce opératoire de l’omphalectomie

Il s’agissait d’une patiente de 42 ans, nulligeste, aux antécédents de myomectomie 5 ans auparavant, qui a consulté pour une tuméfaction ombilicale évoluant depuis 06 mois, devenant violacée et sensible en début de règles avec écoulement de liquide brunâtre, épais en fin de règles. A l’interrogatoire, elle présentait des épisodes de métrorragies et de douleurs pelviennes chroniques, cycliques. Il y’avait pas de notion de dyspareunie ni de cystalgie. L’examen clinique objectivait une masse ombilicale arrondie, de 3 cm de diamètre, noirâtre avec une induration à sa base, sensible à la palpation et une cicatrice d’incision de Pfannenstiel (Figure 1). Les touchers pelviens étaient sans particularité. La biopsie de la masse ombilicale a été réalisée. L’examen histologique de la pièce de biopsie a montré un revêtement épidermique hyperplasique, un derme discrètement inflammatoire et une jonction dermo-hypodermique avec une fibrose cicatricielle, une plage de cellules évocatrice d’un chorion cytogène et une structure glandulaire de type endométrial. IRM du pelvis, pratiquée à la recherche de lésions évocatrices d’endométriose, était sans particularités. L’intervention chirurgicale a été décidée, associant une omphalectomie et une mini-laparotomie exploratrice du pelvis. Une exérèse complète du nodule ombilical a été réalisée avec des limites macroscopiques en zone saine, suivie d’une plastie ombilicale (Figure 2). L’exploration du pelvis par une mini laparotomie n’avait pas objectivé d’autres lésions endométriales. L’examen histologique de la pièce opératoire avait montré un foyer d’endométriose dans le derme et l’hypoderme avec des marges des résections saines. Les suites opératoires étaient simples. Une contraception progestative a été instaurée en postopératoire.

## Discussion

L’endométriose correspond à la présence anormale de tissu endométrial fonctionnel en dehors de la cavité utérine. L’endométriose ombilicale, connue sous le nom de nodule de Villar, est une rare manifestation extra-pelvienne de l’endométriose [4]. Sa fréquence serait de l’ordre de 1%, l’ensemble des localisations de la maladie. Elle touche préférentiellement la femme en période d’activité génitale. Elle est rare avant les ménarches et tend à diminuer après la ménopause [5]. L’endométriose est une maladie chronique, dont la cause est probablement multifactorielle et le mécanisme physiopathologique reste en partie non élucidé. Sur le plan physiopathologique plusieurs théories ont été proposées pour expliquer la genèse de l’endométriose. La théorie de Sampson, elle est actuellement la plus largement acceptée. Selon cette théorie, l’endométriose proviendrait de cellules endométriales viables refluant à travers les trompes pendant les règles et s’implantant sur la surface du péritoine et des organes pelviens [6]. La théorie de Meyer suggère que l’endométriose provient d’un processus métaplasique. Les cellules dérivées de l’épithélium cœlomique subiraient une métaplasie vers les cellules endométriales, sous l’effet de divers facteurs infectieux, toxiques ou hormonaux [7]. La théorie de la métastase lymphatique et vasculaire a proposé que l’endométriose puisse résulter d’une dissémination par voie lymphatique et hématogène des cellules endométriales. La métastase par voie lymphatique jusqu’à des sites distants comme l’ombilic, l’espace rétro péritonéal, des membres inférieurs, est anatomiquement possible du fait d’une communication du système lymphatique entre ces différentes structures et l’endomètre. Dans notre cas, la théorie de Sampson et la métastase par voie lymphatique pourraient expliquer la localisation ombilicale. L’endométriome ombilicale se manifeste par une masse ombilicale douloureuse avec un écoulement sanglant rythmé par le cycle menstruel. Le caractère cyclique qui coïncide avec les menstruations est fondamental et parfois suffisant pour évoquer le diagnostic [8].

Les signes peuvent apparaitre des semaines ou des années après la chirurgie avec un délai moyen de 4,8 ans. L’échographie, le scanner et l’IRM ont été utilisés pour aider au diagnostic. Les aspects ne sont pas spécifiques et dépendent de plusieurs éléments tels que la phase du cycle menstruel, l’importance de la réaction inflammatoire et de la répartition entre les éléments du stroma et ceux glandulaires. L’IRM permet également de confirmer l’existence d’autres localisations, notamment pelviennes [9]. La confirmation du diagnostic repose sur l’examen anatomopathologie. L’étude histopathologie de la pièce opératoire montre les glandes endométriales en position ectopique avec des tubes glandulaires, du chorion cytogène et des fibres musculaires. Le traitement de l’endométriome ombilical est chirurgical. Le gold standard du traitement repose sur une large exérèse de la masse avec des marges de sécurité d’au moins 1cm afin de réduire le risque de récidive. La fermeture de la paroi est réalisée en plans anatomiques et la dépression ombilicale est obtenue à l’aide d’un point de suture par fil résorbable qui attache le derme à l’aponévrose des muscles droits [10, 11]. Une exploration du pelvis par une mini laparotomie sous ombilicale ou par cœlioscopie à la recherche d’autres localisations est sans risque. La prévention en cas de laparotomie est basée sur le lavage abondant de la cavité abdominale et de la cicatrice en fin d´intervention ainsi que le changement de gants pour le temps de fermeture pariétale, alors qu´en cœlioscopie, l´extraction des pièces opératoires dans un sac de protection et le lavage abondant de la cavité pelvienne devraient être systématiques. Ainsi, ces mesures relèvent de la bonne pratique chirurgicale bien que leur bénéfice n´a jamais été démontré [11].

## Conclusion

La localisation ombilicale de l’endométriose est rare. La symptomatologie cyclique dans les suites proches ou lointaines d'une chirurgie gynécologique doit faire évoquer le diagnostic afin d’établir une stratégie thérapeutique adaptée qui repose essentiellement sur la chirurgie.

## Conflits d’intérêts

Les auteurs ne déclarent aucun conflit d'intérêts.
